# Purification of Adeno-Associated Virus (AAV) Serotype 2 from *Spodoptera frugiperda* (Sf9) Lysate by Chromatographic Nonwoven Membranes

**DOI:** 10.3390/membranes12100944

**Published:** 2022-09-27

**Authors:** Jinxin Fan, Eduardo Barbieri, Shriarjun Shastry, Stefano Menegatti, Cristiana Boi, Ruben G. Carbonell

**Affiliations:** 1 Department of Chemical and Biomolecular Engineering, North Carolina State University, Raleigh, NC 27695, USA; 2 Golden LEAF Biomanufacturing Training and Education Center (BTEC), North Carolina State University, Raleigh, NC 27606, USA; 3 Department of Civil, Chemical Environmental and Materials Engineering, DICAM, University of Bologna, Via Terracini 28, 40131 Bologna, Italy; 4 National Institute for Innovation in Manufacturing Biopharmaceuticals (NIIMBL), Newark, DE 19711, USA

**Keywords:** adeno-associated virus (AAV), viral vector purification, nonwovens, membrane adsorber, anion exchange, cation exchange, membrane chromatography

## Abstract

The success of adeno-associated virus (AAV)-based therapeutics in gene therapy poses the need for rapid and efficient processes that can support the growing clinical demand. Nonwoven membranes represent an ideal tool for the future of virus purification: owing to their small fiber diameters and high porosity, they can operate at high flowrates while allowing full access to target viral particles without diffusional limitations. This study describes the development of nonwoven ion-exchange membrane adsorbents for the purification of AAV2 from an Sf9 cell lysate. A strong anion-exchange (AEX) membrane was developed by UV grafting glycidyl methacrylate on a polybutylene terephthalate nonwoven followed by functionalization with triethylamine (TEA), resulting in a quaternary amine ligand (AEX-TEA membrane). When operated in bind-and-elute mode at a pH higher than the pI of the capsids, this membrane exhibited a high AAV2 binding capacity (9.6 × 10^13^ vp·mL^−1^) at the residence time of 1 min, and outperformed commercial cast membranes by isolating AAV2 from an Sf9 lysate with high productivity (2.4 × 10^13^ capsids·mL^−1^·min^−1^) and logarithmic reduction value of host cell proteins (HCP LRV ~ 1.8). An iminodiacetic acid cation-exchange nonwoven (CEX-IDA membrane) was also prepared and utilized at a pH lower than the pI of capsids to purify AAV2 in a bind-and-elute mode, affording high capsid recovery and impurity removal by eluting with a salt gradient. To further increase purity, the CEX-IDA and AEX-TEA membranes were utilized in series to purify the AAV2 from the Sf9 cell lysate. This membrane-based chromatography process also achieved excellent DNA clearance and a recovery of infectivity higher that that reported using ion-exchange resin chromatography.

## 1. Introduction

Giulio Sarti has made tremendous contributions to membrane science and technology, covering a wide range of areas such as gas separations, membrane distillation, and bioseparations. With this work we honor his exemplary career, and the great impact he has had on numerous students and professionals over the years. Giulio Sarti is the thread that connects the senior authors of this manuscript, and we have all benefitted from his knowledge and expertise for many years as colleagues, collaborators, students, and friends.

Gene therapies offer the promise to treat or cure a wide variety of diseases caused by congenital immune deficiencies, such as hereditary blindness, hemophilia, leukemia, cardiovascular disease, fat metabolism disorders, and infectious diseases [1,2,3,4,5]. Among the various viral vectors known to date [6,7], adeno-associated virus (AAV) vectors are viewed as a highly versatile and safe vehicle for gene delivery, owing to their high tissue penetration as well as low immunogenicity, hepatotoxicity, and genotoxicity [6,8]. Three AAV vector-based treatments have gained regulatory approval and more than 150 clinical trials are ongoing [6,9].

The clinical success of AAV therapeutics has spurred a keen interest in developing efficient and robust manufacturing processes for these vectors [10,11,12,13]. AAV vectors are recombinantly expressed either in human embryonic kidney 293 cells (HEK293) or insect cells, specifically *Spodoptera frugiperda* (Sf9) cell lines. The vectors must then be purified and concentrated using a series of downstream steps that eliminate host cell proteins (HCPs), double-stranded DNA (dsDNA) and other oligonucleotide fragments, and viral capsids that are empty or contain fragmented genes [8,10,11]. Despite the significant advancements in the design and engineering of host cells and bioreactors for large-scale virus cultivation [14], downstream capture and purification steps are costly and time-consuming [9,15].

Ultracentrifugation-based virus separation can be used for preparing small batches of pure pre-clinical samples but is not applicable to large-scale manufacturing due to low capacity and productivity and high energy consumption [12,16,17]. Chromatographic column approaches provide higher resolution and throughput and are well established in commercial vector manufacturing [17,18]. The majority of resin-based chromatographic media, however, exhibit low viral particle dynamic binding capacities, since the pore diameter of the resins, usually less than 50–100 nm, may limit adsorption and pore diffusion of viral particles, whose diameters range from tens to hundreds of nanometers [19]. As a result, the capture of viral particles on resin beads are restricted mainly to the bead surface, resulting in poor ligand utilization [20,21].

Diffusional limitations can be overcome by increasing the residence time (RT) in the column, although this results in longer process times [22,23] and increases the risk of aggregation and degradation of viral particles [9,24]. Diffusional limitations can also be addressed by decreasing the particle size, resulting in high pressure drops that limit the practical length of the column [25].

To address such issues, membrane adsorbers and monoliths with large pore sizes have also been used for viral vector purification [26,27]. These separation media usually exhibit higher viral particle dynamic binding capacities than resins, especially at shorter residence times [28,29]. Some membrane adsorbers may be used as single-use disposable devices, eliminating costly and time-consuming cleaning-in-place and validation steps [20,25,30]. Because of these characteristics and ease of scale-up, membrane chromatography is ideally suited for the purification of viral particles [19,21]. Nestola et al. recently reported that both the ligand density and porous structure of cast polymeric anion-exchange membranes play critical roles in the separation of large viruses, including recombinant baculovirus and adenovirus serotype 5 (Ad5) [25]. McNally et al. optimized the buffer conditions in a bind-and-elute anion-exchange step using Mustang Q membranes to recover retrovirus with high purity [31]. Ghosh et al. demonstrated an efficient Ad5 separation using a laterally fed holder packed with Sartobind Q membranes, showing that this device outperformed a commercial radial flow system [20]. The cast membranes used in these studies, however, are not amenable to single-use applications [32,33].

Because of the low cost of large-scale production of nonwoven fabrics, fiber-based nonwoven membranes show great potential for development of single-use membrane devices. Nanofiber membranes feature high surface areas and porosities, which provide high binding capacities and fast flow rates for increased productivity [34,35]. Only a few studies have focused on the use of fiber-based membrane adsorbents for viral vector purification. Turnbull et al. fabricated cellulose nanofiber-based nonwovens by electrospinning and functionalized them with quaternary amine ligands to capture adenovirus [28]. By optimizing the ligand density, the nonwoven outperformed other membrane adsorbents and monoliths in terms of infective capsid recovery [28], although the purity of the samples was not reported. Ruscic et al. used the same membrane adsorbent for purification of lentiviral vector and achieved a 90% yield of functional vector [34].

Our team has developed a class of nonwoven membrane adsorbers for the chromatographic purification of biologics, comprising polybutylene terephthalate (PBT) nonwoven fabrics grafted with polyglycidyl methacrylate (GMA) layers that can be functionalized with a variety of ligands [36,37,38]. These membranes exhibit excellent flow permeability, high binding capacity and selectivity towards proteins, and can be operated in bind-and-elute mode for product capture or flow-through mode for product polishing. To date, there are no available reports on the purification of AAV using membranes based on fibrous nonwoven materials. This study is the first to utilize nonwoven ion-exchange membranes to demonstrate a process for rapid and efficient separation of AAV serotype 2 (AAV2) from a cell culture lysate. For this purpose, nonwoven membranes UV grafted with glycidyl methacrylate (GMA) were functionalized with (i) triethylamine (TEA) to form a quaternary ammonium anion-exchange membrane (AEX-TEA membrane), and (ii) iminodiacetic acid (IDA), a carboxylic acid ligand, to form a cation-exchange membrane (CEX-IDA membrane). While the AEX-TEA membrane is a new anion exchanger, the CEX-IDA membrane was previously developed for the purification of monoclonal antibodies [38], although it was never applied in the purification of viral vectors. 

The binding and elution of AAV2 on the AEX-TEA membrane were initially characterized in a non-competitive mode using pure AAV2; subsequently, the membrane was used to purify AAV2 from an Sf9 cell lysate by a bind-and-elute capture step, using a commercial Sartobind Q membrane as a control. To increase product purity, we implemented a two-step membrane chromatography process using a cation-exchange bind-and-elute step using the CEX-IDA membrane followed by a second bind-and-elute step using the AEX-TEA membrane. Both operations were conducted at short residence times (i.e., sample loading at 1 min RT and equilibration, washing and elution steps at 0.2 min RT). This two-step membrane process achieved a high overall infective capsid recovery of 76.0% and high removal of host cell proteins (LRV ~ 3.3) and DNA (LRV ~ 4.2).

## 2. Experimental Procedure

### 2.1. Materials

PBT nonwovens were obtained from Macopharma (Tourcoing, France). The nonwoven fabric has a basis weight of 55 g/m^2^ and a thickness of approximately 200 µm. The average fiber diameter of the nonwoven is 3 µm, the average pore size is 8 µm, and the overall porosity is approximately 80%. Glycidyl methacrylate was purchased from Reagent World (Ontario, CA, USA). Benzophenone, benzoyl peroxide (BPO), triethylamine (TEA), sulfuric acid, butanol, sodium chloride (NaCl), hydrochloride acid (HCl), sulfuric acid, tris(hydroxymethyl) aminomethane (Tris), sodium acetate, acetic acid, sodium hydroxide (NaOH), and citric acid (CA) were purchased from Millipore Sigma (St. Louis, MO, USA). Sartobind Q pico 0.08 mL was purchased from Sartorius (Goettingen, Germany). CEX-IDA membrane was fabricated following the method developed in [38]. The clarified Sf9 cell lysate and a solution of pure AAV2 carrying a green fluorescent protein (GFP) transgene were purchased from Virovek (Hayward, CA, USA). AAV2 has a capsid of about 23–28 nm diameter, and the reported pI of the full capsid is 5.9 [39]. This is different from the pI of the empty capsids which is 6.3 [39]. The properties of the feed solutions used in this study are listed in Table 1. The main impurity protein in the Sf9 cell lysate is the expressed green fluorescent protein (GFP, 26 kDa, pI of 5.5 [40]), as confirmed by the SDS-PAGE analysis. The Pierce™ Bradford assay kit used to measure the total protein concentration was obtained from Thermo Fisher Scientific (Pittsburgh, PA, USA). AAV2 titration ELISA 2.0R was purchased from American Research Products (Waltham, MA, USA). The sodium dodecyl sulfate poly-acrylamide gel electrophoresis (SDS-PAGE) kit was purchased from Bio-Rad (Hercules, CA, USA). The SilverQuest™ silver staining kit and the Quant-iT™ PicoGreen™ dsDNA assay kit were bought from Fisher Scientific (Fairlawn, NJ, USA). The above-listed kits were used following the instructions from manufacturers.

### 2.2. Preparation of Ion-Exchange Nonwovens

The PBT nonwovens were modified with polyGMA by UV grafting according to our prior work [36,37]: 1 mL of GMA grafting solution comprising BP at 13.7 mg/mL dissolved in 20% *v*/*v* GMA in butanol was sprayed on the PBT nonwoven, which was sandwiched between two glass slides and irradiated with UV light (λ = 365 nm; intensity: 5 mW/cm^2^) using an EN-180, lamp (Spectronics Corporation, Westbury, NY, USA). A grafting time of 19 min was used for all the membranes in this study to obtain grafting weight gains of 20% (calculated as the weight gain of the sample after GMA grafting divided by the nonwoven weight of the sample before grafting). The polyGMA-grafted PBT nonwovens were washed thoroughly in THF and methanol under sonication, and dried in a vacuum oven at room temperature. The AEX-TEA membranes were prepared by incubating the polyGMA-grafted PBT nonwovens (50 mm × 70 mm) in 50 mL of 10% *v*/*v* TEA in water. The reaction temperature (20–60 °C) and reaction time (4.0–24.0 h) were optimized during membrane preparation. The unreacted epoxy groups were hydrolyzed in 0.1 M sulfuric acid at 50 °C for 16 h, after which the membranes were washed and dried as described above. A scanning electron microscope (SEM, Hitachi S-3200 N, Hitachi High-Tech, Schaumburg, IL, USA) was used to image the membrane structure after preparation. The CEX-IDA membrane was prepared following the process established in published work [38].

### 2.3. Dynamic AAV2 Binding Capacity of AEX-TEA Membranes in Non-Competitive Conditions (Pure AAV2)

A stack of 6 layers of AEX-TEA membranes was placed on an Omnifit column (diameter: 10 mm, height: 0.12 cm; Diba Industries, Inc., Cambridge, UK) to obtain a total membrane column volume of 0.1 mL. The packed column was installed on an ÄKTA™ pure system (Cytiva, Marlborough, MA, USA) for dynamic binding studies of AAV2 binding. The membranes were equilibrated in equilibration buffer (50 mM Tris HCl, pH 8.0) until the UV baseline was stable, and loaded with 10 mL of standard AAV2 solution with a titer of 1 × 10^12^ AAV2/mL (Table 1) at pH of 7.4 and conductivity of 4–5 mS/cm. To promote binding of the capsids, 10 mL of feed solution was adjusted from pH 7.4 to pH 8.0 by 0.1 M NaOH. The capsid loading amount during the experiment was 1 × 10^14^ capsids per mL of membrane volume. The membranes were then washed with 10 mL of 50 mM Tris HCl at pH 8.0, and the elution was carried out by sequential NaCl step gradients (5 mL for each step) with concentrations ranging from 0.1 to 2.0 M. The residual bound capsids were stripped using 0.1 M NaOH. All chromatographic steps were conducted at a residence time (RT) of 0.2 min, except the sample loading, which was conducted at an RT of 1 min. The corresponding chromatogram was analyzed by UV peak area calculation to determine the number of capsids in the collected fractions. The number of capsids in the various chromatographic fractions were calculated as follows:

$$\mathrm{Number}\;\mathrm{of}\;\mathrm{capsids}=\frac{A_{\mathrm{Specific}\;\mathrm{fraction}}}{A_{\mathrm{Total}\;\mathrm{area}}}\times \;\mathrm{Number}\;\mathrm{of}\;\mathrm{loaded}\;\mathrm{capsids}$$

### 2.4. AAV2 Purification from Sf9 Cell Lysate with AEX-TEA Membranes

Two devices were compared for AAV2 purification from an Sf9 lysate: an Omnifit column packed with 6 AEX-TEA membranes (0.1 mL) and a Sartobind Q pico unit of 0.08 mL membrane module. To promote capsid binding, the Sf9 lysate solution was adjusted to pH 8.0 and a conductivity of 4 mS/cm via 20-fold dilution with 50 mM Tris HCl pH 8.0. After dilution, the total capsid titer was 5.5 × 10^11^ capsids/mL, on the same scale of the pure AAV2 sample (1.0 × 10^12^ capsids/mL) described in Section 2.3 (Table 1). The membranes were equilibrated in 50 mM Tris HCl pH 8.0 and loaded with diluted Sf9 lysate at a ratio of 2.5 × 10^13^ capsids per mL of membrane at an RT of 1 min: specifically, 4.5 mL and 3.6 mL of diluted lysate were loaded to the AEX-TEA and Sartobind Q membranes, respectively. After washing with 50 mM Tris HCl pH 8.0, the bound capsids were eluted by running a step gradient with different NaCl concentrations from 0.1 to 1 M (7 mL for each step) to find the optimal elution conditions. The equilibration, washing, and elution steps were conducted at an RT of 0.2 min. All chromatographic fractions were collected for sample analysis as described in Section 2.6.

### 2.5. Purification of AAV2 from Cell Lysate by a Two-Step Membrane Chromatography Process

A stack of 12 CEX-IDA membranes were packed into an Omnifit column holder (diameter: 10 mm; volume: 0.25 mL). To optimize capsid binding, three binding buffers were prepared comprising 50 mM acetate at pH of 5.0, 5.5, and 6.0, and utilized to equilibrate the membrane column and to dilute the Sf9 lysate 20-fold to a conductivity of 3.0 mS/cm and a capsid titer of 1 × 10^11^ capsids/mL. Following filtration with a 0.45 μm membrane, a volume of 4.5 mL of diluted Sf9 lysate was loaded on the column, corresponding to a loading ratio of 1.8 × 10^12^ capsids per mL of membrane volume, at an RT of 1 min. After washing the membranes with 7.5 mL of binding buffer, the bound capsids were eluted by 5.0 mL elution buffer (1 M NaCl added to the corresponding binding buffer). Finally, the membranes were cleaned with 5.0 mL of 0.1 M citric acid (CA) at pH 2.0. The equilibration, washing, elution, and cleaning steps were conducted at 0.2 min RT. Once the optimal binding pH was identified, a sequential step gradient of 0.1, 0.2, 0.3, and 1 M NaCl was employed to optimize capsid elution. Based on the results obtained, the binding and elution buffers were adjusted to 0.1 M NaCl and 1 M NaCl, respectively, in 50 mM acetate at pH 5.0.

The 1 M NaCl eluate from the CEX-IDA membrane was adjusted to an AAV2 titer of 1 × 10^11^ capsids/mL, pH 8.0, and ~9 mS/cm via dilution with 50 mM Tris HCl pH 8.0 and pH adjustment by 0.1 M NaOH. A stack of 24 AEX-TEA membranes were packed into an Omnifit column holder (diameter: 10 mm; volume: 0.5 mL) and equilibrated with 50 mM Tris HCl at pH 8.0. The AAV2 solution was loaded onto the column at a ratio of 2.4 × 10^11^ capsids per mL of membrane and RT of 1 min. After washing the membranes with 50 mM Tris HCl at pH 8.0, the bound capsids were eluted by a sequential elution of 0.1 M, 0.2 M, and 1 M NaCl (7.5 mL for each step) in Tris HCl buffer at pH 8.0. The equilibration, washing, and elution steps were conducted at an RT of 0.2 min. To study the impact of RT, this two-step membrane process was repeated using an RT of 0.1 min for sample loading, while 0.2 min RT was kept for other chromatographic steps. All chromatographic fractions were collected for sample analysis as described in Section 2.6.

### 2.6. Sample Analysis

#### 2.6.1. Total Protein Concentration, AAV2 Capsid (Full and Empty) Concentration, and dsDNA Concentration Measurement

The mass of total protein in the fractions was measured via Bradford assay. The total concentration of AAV2 capsids was determined by ELISA according to instructions provided by the manufacturer. dsDNA concentration was measured by Quant-iT™ PicoGreen™ dsDNA assay kit.

#### 2.6.2. Viral Genome (VG) Quantification by qPCR

The viral genome content was quantified by qPCR: samples were initially treated with 20 U/µL of DNase (Invitrogen, Waltham, MA, USA) for 1 h at 37 °C followed by DNAse deactivation at 95 °C for 20 min; the samples were then treated with 10 µg/µL Proteinase K (Invitrogen, Waltham, MA, USA) for 1 h at 60 °C, followed by deactivation at 95 °C for 10 min; the samples were mixed with GFP forward and reverse primers (AGC AAA GAC CCC AAC GAG AA and GGC GGT CAC GAA), Taqman TAMRA probe (CGC GAT CAC ATG GTC CTG G), and Q-PCR expression mix (Invitrogen, Waltham, MA, USA); finally, qPCR was performed on a Chai PCR machine (Chai, Santa Clara, CA, USA). The standard curve was constructed via serial dilutions of known concentration of plasmid (Virovek, Hayward, CA, USA).

#### 2.6.3. Transduction Activity Assay

The transduction activity of AAV2 products was quantified using HT1080 cells (ATCC, Manassas, VA, USA): the cells were cultured in DMEM media supplemented with 10% *v/v* FBS (Corning life sciences, NY, USA) at 37 °C and 5% CO_2_; after reaching 70–80% confluence, the cells were treated with trypsin and collected by centrifugation, and transferred to a 96-well plate at 6000 cells/well; after 12 h, the cells were incubated with AAV2 samples diluted (1:100) in DMEM and FBS mixture with added 8.0 µg/mL of polybrene (Sigma, St. Louis, MO, USA). After 24 h incubation, the media were removed and substituted with DMEM and FBS mixture; the growth media weree removed after 48 h, and the cells were detached using a mixture of 75% TrypLE and 25% DPBS (Gibco, Waltham, MA, USA); finally, the number of cells expressing GFP was measured by using a Beckman Coulter CytoFLEX (Beckman Coulter Life Sciences, Brea, CA, USA), and the transducing units/mL defined as the number of GFP-expressing cells per mL of injected AAV2 sample. The infective recovery was determined by comparing the total transducing units in the purified samples and that in the feed.

#### 2.6.4. Capsid Imaging via Transmission Electron Microscopy (TEM)

The aliquots of AAV2 samples were dropped on TEM grids, stained with 1% NanoW stain (Nanoprobes, Yaphank, NY, USA), and dried overnight; the stained capsids were imaged using a semi-automated MiniTEM system (Vironova, Stockholm, Sweden).

## 3. Results and Discussion

### 3.1. Preparation of AEX-TEA Membrane

**Figure 1:**
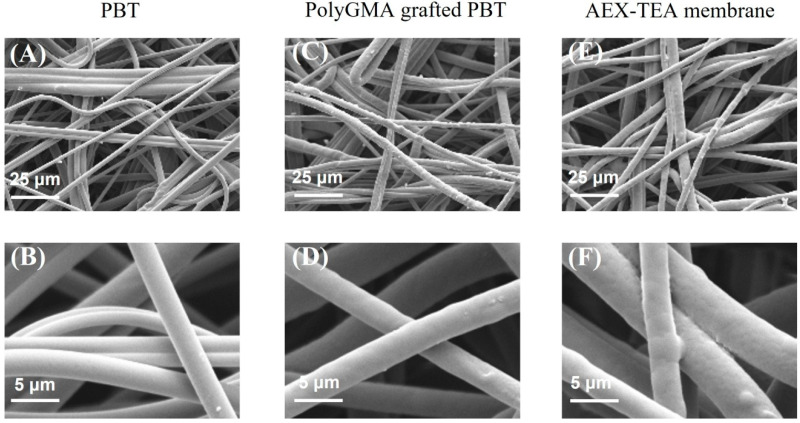
SEM images of the membrane surface for (**A**,**B**) original PBT membrane, (**C**,**D**) polyGMA grafted membrane, and AEX-TEA membrane (**E**,**F**) at different magnifications.

Ion-exchange chromatography is a key technology for virus purification: the difference in electrostatic charge between gene-carrying capsids and process-related impurities present in the feedstock makes ion exchangers effective tools for virus capture [8,41]. Additionally, gene-carrying capsids feature a distinctive negative charge, which enables the use of anion-exchange chromatography (AEX) to isolate them from empty or incorrectly loaded capsids [18,42]. To leverage the advantages of nonwoven ion-exchange membranes for the chromatographic purification of AAVs, we developed a nonwoven quaternary amine AEX membrane by coupling of TEA to PBT fibers coated with a UV-grafted polyGMA layer. The ligand coupling temperature and time were optimized to promote binding capacity. As shown in Figure S1A,B, under the optimal condition, i.e., reacting at 40 °C for 8 h, the resultant AEX-TEA membrane exhibited a remarkable value of static binding capacity of bovine serum albumin (BSA), namely 282.0 mg per g of membrane; furthermore, its dynamic binding capacities measured at residence times (RT) from 0.1–2.0 min were found to be approximately 44.0 mg/mL (Table S1 and Figure S2). The SEM images in Figure 1 demonstrate that the fiber structure remained intact after ligand coupling and exhibit uniform, conformal GMA coatings on the fibers. The large inter-fiber distance of 3–40 μm, shown in Figure 1E, explains the good flow permeability of the membranes: specifically, regression of the pressure drop vs. flow rate data using Darcy’s law resulted in a permeability of 3.5 × 10^−13^ m^2^ (Figure S3), a value that is significantly higher than values reported for commercial Q resins (particle diameters of 50–90 μm), which range from 1.1 × 10^−14^ to 2.2 × 10^−14^ m^2^ [43]. Most significantly, the AEX-TEA membrane also showed minimal non-specific binding, resulting in excellent selectivity in fractionating BSA from a BSA/immunoglobulin G (IgG) mixture, achieving a BSA purity of 98% (Figure S4). Capitalizing on this behavior, we studied the capture of pure AAV2 and the purification of AAV2 from a clarified Sf9 lysate using the AEX-TEA membrane in bind-and-elute mode.

### 3.2. Pure AAV2 Bind-and-Elute Experiment with AEX-TEA Membrane

**Figure 2:**
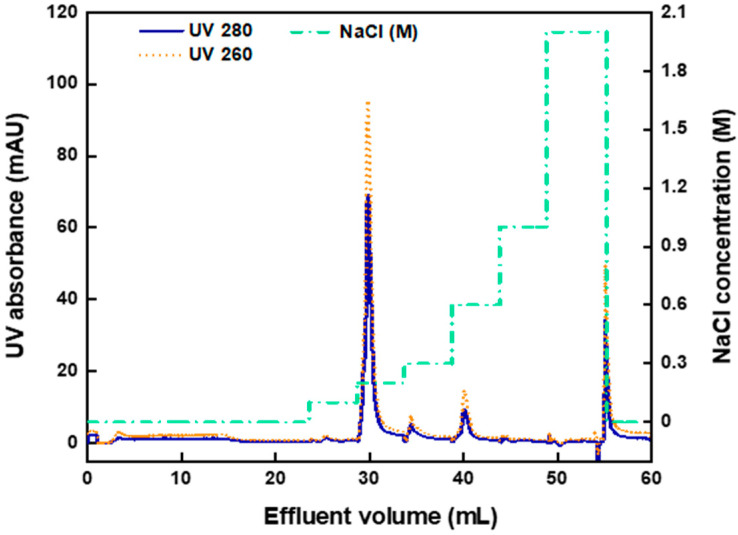
Chromatogram of pure AAV2 (full capsid) binding and elution by AEX-TEA membrane operated in bind-and-elute mode at the RT of 1 min. The stepwise elution gradient was operated at a steady pH 8 by implementing NaCl concentrations of 0.1, 0.2, 0.3, 0.6, 1, and 2 M, then stripped by 0.1 M aqueous NaOH.

Figure 2 presents the chromatogram of pure AAV2 binding and elution by the AEX-TEA membrane. The capture step was performed at pH 8.0, where the capsids are negatively charged (*note:* the values of isoelectric points (pI) of full and empty capsids are 5.9 and 6.3, respectively) and can be effectively adsorbed by the cationic quaternary ammonium ligands. There is a broad and small UV signal that appeared in the flowthrough volume from 3 to 17 mL, corresponding to roughly 4% of the amount of the loaded capsids. As most AAV2 capsids were captured by the membrane, a binding capacity of approximately 9.6 × 10^13^ capsids/mL of column was calculated. As far as we are aware, there are no reports in the literature of binding pure AAV2 capsids on anion-exchange chromatography adsorbents, although the values of AAV binding from cell culture supernatants on adsorbents with quaternary ammonium ligands are generally substantially lower (1 × 10^12^–1.5 × 10^13^ capsids/mL) [44,45,46,47].

The majority (87%) of the loaded capsids were eluted at 0.2 M NaCl, as shown in Figure 2. Minor peaks appeared when the NaCl concentration was increased to 0.3 M and to 0.6 M, which may be caused by capsids or capsid aggregates more strongly bound to the membrane. Approximately 10% of the loaded capsids were lost due to irreversible binding to the membrane, as they did not elute even at 2.0 M NaCl and were ultimately stripped using an aqueous 0.1 M NaOH solution. This could be due to capsid aggregation, loss of integrity, or entrapment in the adsorbent structure, as reported in several studies on viral vector purification by ion-exchange chromatography [28,48,49].

### 3.3. AAV2 Separation from Cell Lysate with Anion-Exchange Membranes

**Figure 3:**
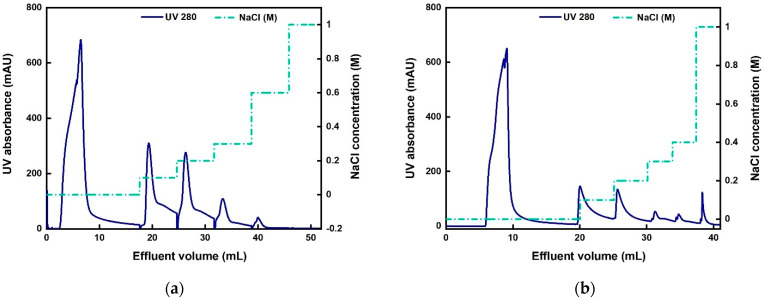
Chromatograms of AAV2 purification from the Sf9 lysate via (**a**) AEX-TEA membrane and (**b**) Sartobind Q membrane conducted at a binding RT of 1 min and a pH of 8.0. Step elution gradients of 0.1, 0.2, 0.3, 0.6, and 1 M NaCl were used for the AEX-TEA-membrane, while 0.1, 0.2, 0.3, 0.4, and 1 M NaCl were used for the Sartobind Q membrane.

**Figure 4:**
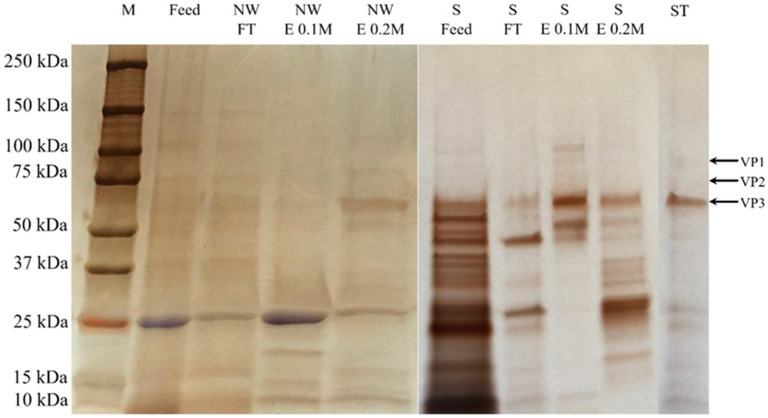
SDS-PAGE analysis of AAV2 purification from the Sf9 lysate via AEX-TEA nonwoven membrane (“NW”) and Sartobind Q membrane (“S”) conducted at a binding residence time (RT) of 1 min and steady pH of 8.0. M: marker; FT: flow-through; E 0.1 M: eluate by 0.1 M NaCl elution buffer; E 0.2 M: eluate by 0.2 M NaCl elution buffer; ST: AAV2 standard (full capsids).

The AAV2 separation performance of the AEX-TEA membranes was evaluated using an Sf9 lysate containing expressed AAV2 and compared to results using a commercial Sartobind Q membrane. The lysate was conditioned to pH 8.0 via dilution with equilibration buffer (*note:* dilution was adopted in lieu of diafiltration to avoid loss of AAV2 titer). Following the excellent product recovery (~87%) achieved with the stepwise elution gradient of pure AAV2, a similar elution strategy was adopted for isolating AAV2 from the lysate. The chromatograms obtained using the two anion-exchange membranes are shown in Figure 3, and the quantitative results and protein characterization by SDS-PAGE are reported in Table 2 and Figure 4. 

Figure 3a shows a large peak in the flow-through fraction which contained only 1.3% of the loaded total capsids. This large flow-through peak consists primarily of a large amount of GFP present in the supernatant, as well as other host cell proteins. GFP has a pI of 5.5 and it can bind to the membrane at pH 8.0. The amount of GFP in the feed is so high that it exceeds the binding capacity of the membrane. Notably, the capsids recovered in the elution fractions collected at 0.1 and 0.2 M NaCl accounted for 87.9% of the loaded capsids, a number that closely mimics the results obtained with the pure AAV2 samples. A similar elution profile was observed with the Sartobind Q membrane, as shown in Figure 3b, although in this case 25.8% of loaded capsids were lost in the flow-through fraction, and 59% of the total capsids were recovered in the 0.1–0.2 M NaCl elution fractions. These results indicate that the total number of capsids bound per mL of membrane was higher in the AEX-TEA membrane (2.4 × 10^13^) than in the Sartobind Q membrane (1.9 × 10^13^).

The higher binding capacity for the AEX-TEA membrane may be attributed to its grafted binding layer, which provides a significant volume for product capture. The intrinsic surface area of the original PBT nonwoven (0.86 m^2^/g) is similar to that of the Sartobind Q membrane (0.90 m^2^/g). As shown in previous work, the grafted layer thickness of our nonwoven membranes at 20% weight gain of GMA is of the order of 2000 Å or more. This allows the binding of viruses within the flexible polyelectrolyte layer at low ionic strength, and the subsequent release of them at high ionic strength. It should be noted (Table 2) that the ligand density in the AEX-TEA membrane and the Sartobind Q membrane (600 µm/g vs. 553 µm/g) only differ by approximately 10%, but the larger ligand density for the nonwoven fabric might also play a role in increasing the binding capacity.

The main impurity in the lysate, namely the GFP produced by the Sf9 cells expressing AAV2, was also detected in the 0.1–0.2 M NaCl elution fractions and likely accounted for part of the area of the elution peaks. As evident in the SDS-PAGE analysis (Figure 4), most of the capsids were eluted from the AEX-TEA membrane at 0.2 M NaCl, and from the Sartorius Q membrane at 0.1 M NaCl, indicating stronger binding in the nonwoven membrane. As shown in Table 2, the concentration of the eluted capsids at 0.2 M NaCl in the AEX-TEA membrane (3.3 × 10^11^ capsids/mL) was greater than the concentration of eluted capsids from the Sartorius Q membranes at 0.1 M NaCl (1.9 × 10^11^ capsids/mL). The concentrations of non-capsid proteins in the elution fraction were lower in the AEX-TEA (4.4 µg/mL) than in the Sartorius Q membrane (5.5 µg/mL). In contrast, the Sartobind Q membrane had a ratio of viral genomes (VG) to total capsids (full and empty) in the elution fraction (0.4) which is almost double that obtained with the AEX membrane (0.2), indicating a higher selectivity for full over empty capsids. In summary, the AEX-TEA membrane exhibited higher capsid binding capacity, recovery, and protein removal performance (1.8 LRV vs. 1.5 LRV) during separation of AAV2 from the Sf9 lysate than the Sartobind Q membrane, although the Sartobind Q membrane exhibited a better performance in separating full from empty capsids.

### 3.4. Two-Step Purification of AAV2 by Membrane Chromatography

**Figure 5:**
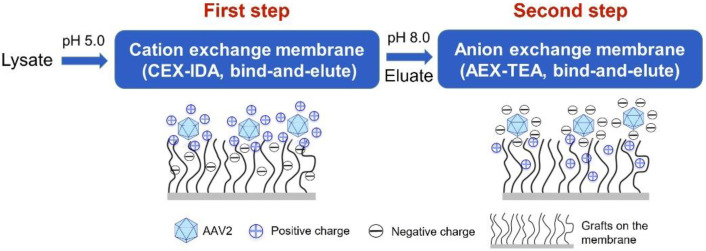
Schematic diagram of two-step purification of AAV2 from Sf9 lysate.

In a recent study, we reported a cation-exchange membrane functionalized with iminodiacetic acid (CEX-IDA membrane) with a high protein-binding capacity and selectivity for the purification of a monoclonal antibody from cell culture fluid [38]. We resolved to evaluate the performance (i.e., purity and yield of AAV2) of a process that utilizes two orthogonal bind-and-elute steps, one cation exchange and one anion exchange, both using membrane adsorbents. The expectation was that, in a first step conducted at a pH lower than the pI of AAV2, the CEX-IDA membrane would capture the capsids while removing unbound negatively charged proteins, while in the second step at a pH higher than the pI of AAV2, the AEX-TEA membrane would concentrate the capsids and remove residual positively charged proteins (Figure 5). To promote AAV2 capture by the CEX-IDA membrane, the process was conducted at constant values of pH 5.0, 5.5, and 6.0, where the AAV capsids display net positive charges and are efficiently adsorbed by the negatively charged carboxyl (pKa: ~3–4 [52,53]) ligands (note: as before, the feedstocks were equilibrated to the corresponding pH via dilution to prevent AAV loss upon diafiltration). The binding step for the AEX-TEA membrane was carried out at pH 8.0, as shown in Section 3.3. The resultant chromatograms are shown in Figures S5–S7 and the quantitative results are listed in Table S2.

As anticipated, the viral particles did not bind to the CEX-IDA membrane at pH 6.0, as this pH is very close to the pI of AAV2 capsids. Upon lowering the pH to 5.5 and 5.0, 76% and the totality of the loaded capsids were captured, respectively, as the binding strength increased with the lower pH, inducing a positive charge to the capsids. 

**Figure 6:**
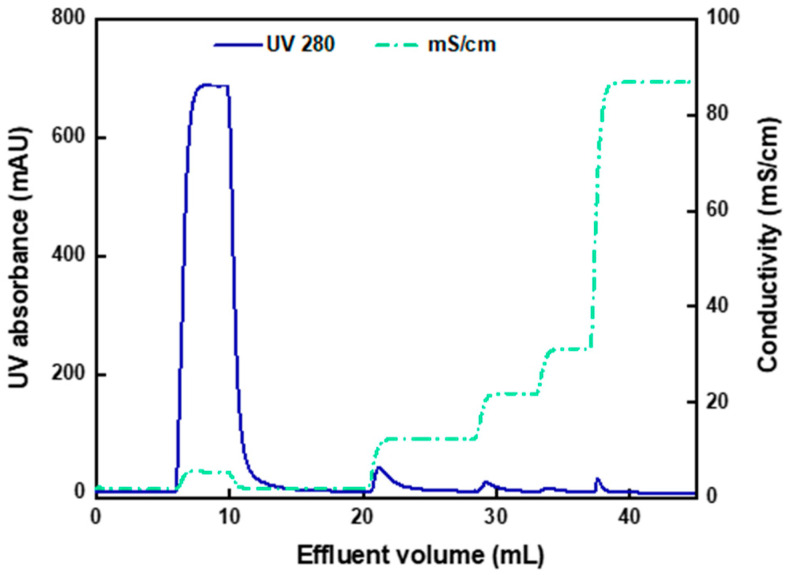
Chromatogram of AAV2 purification from the Sf9 lysate using a 0.25 mL column packed with CEX-IDA membranes at a binding RT of 1 min and pH of 5.0, with step gradients of 0.1, 0.2, 0.3, and 1 M NaCl.

Accordingly, pH 5.0 was used in the subsequent series of experiments performed to study the influence of elution gradients. We took steps to minimize the required time for this bind-and-elute step at pH 5 and introduced a fast equilibration to pH 7.4 (adjusted by 0.1 M NaOH once the eluate was collected) to minimize any potential effects of pH on the viral integrity. When using a sequential elution gradient of 0.1, 0.2, 0.3, and 1 M NaCl at pH 5.0, as shown in Figure 6, no AAV2 capsids were detected in the fraction eluted at 0.1 M NaCl (Table S3), suggesting that the salt screening effect of 0.1 M NaCl was insufficient to release the viral particles; conversely, nearly 84.4% of the loaded viral particles were distributed in the fractions eluted at 0.2, 0.3, and 1 M NaCl. Consequently, the equilibration and elution buffers were adjusted to 0.1 M and 1 M NaCl, respectively.

**Figure 7:**
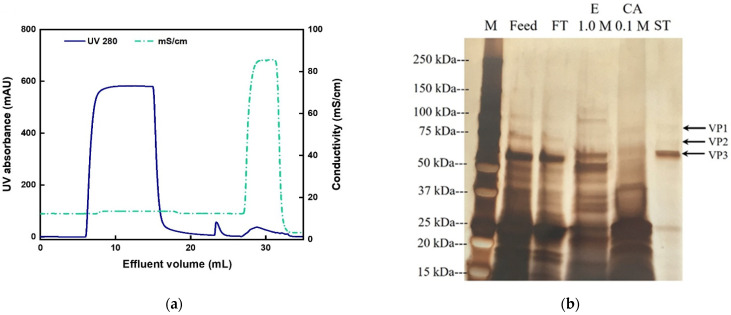
AAV2 separation by CEX-IDA membrane with a step elution of 1 M NaCl. Equilibration buffer: 20 mM acetate buffer at pH 5.0 with 0.1 M NaCl, RT = 1 min, membrane volume = 0.25 mL. (**a**) Chromatogram; (**b**) corresponding SDS-PAGE. M: marker; FT: flow-through; E 1 M: elution with 1 M NaCl buffer; CA 0.1 M: 0.1 M citric acid, pH 2; ST: standard pure AAV2.

The chromatogram and SDS-PAGE analysis of the collected fractions are reported in Figure 7. There were no capsids detected in the flow-through in Figure 7. As shown in Table 3, the values of AAV2 recovery based on ELISA and transduction assay were 87.3% and 88.1%, respectively, with impurity protein removal of 1.1 LRV and DNA removal of 1.5 LRV. As observed in Figure 7b, a large fraction of the Sf9 host cell proteins was found in the flow-through and the cleaning step (0.1 M citric acid) fractions.

**Figure 8:**
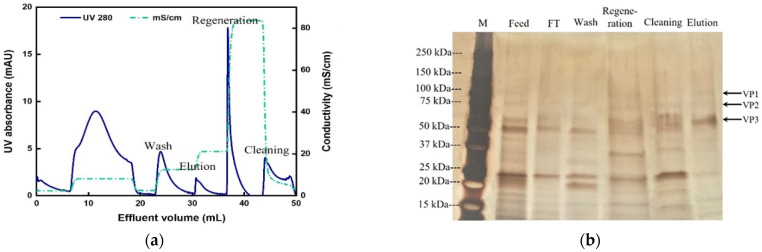
Results of AAV2 purification (second step following CEX-IDA) by AEX-TEA membrane; pH: 8.0, RT: 1 min, membrane volume: 0.47 mL. (**a**) Chromatogram; (**b**) corresponding SDS-PAGE. M: marker; FT: flow-through; Wash: 0.1 M NaCl buffer; Regeneration: 1 M NaCl buffer; Cleaning: 0.1 M citric acid; Elution: 0.2 M NaCl buffer for recovering AAV2.

As shown in Figure 5, the elution stream obtained from the CEX-IDA membrane (first step) was adjusted to pH 8.0 and a conductivity of ~9 mS/cm and loaded onto the AEX-TEA membranes (second step) to polish the AAV2 products. Following on the work presented in Section 3.3, pH 8.0 buffers with NaCl concentration of 0.1 M NaCl (for washing), 0.2 M NaCl (for elution), and 1.0 M NaCl (for regeneration) were used. The resulting chromatogram and the corresponding SDS-PAGE analysis of the collected fractions from the AEX-TEA membrane are reported in Figure 8.

**Figure 9:**
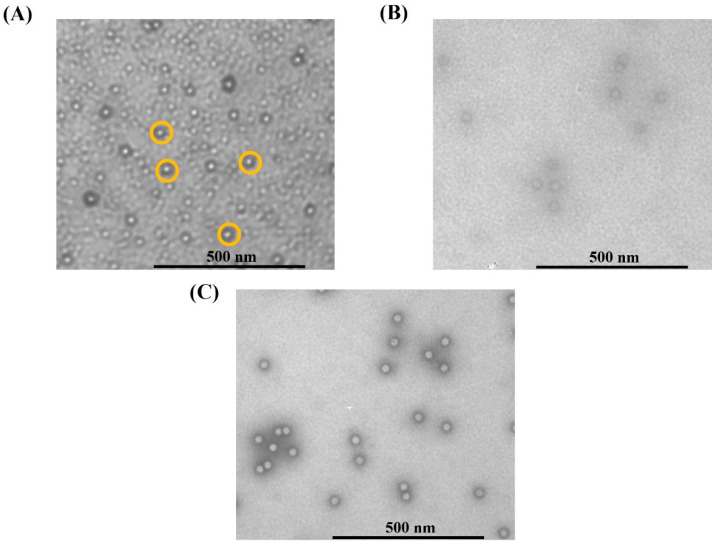
TEM imaging of (**A**) feed, the purified AAV2 after two-step membrane process (**B**), and the standard full AAV2 capsids (**C**). The yellow cycles indicate viral particles.

Several bands could be observed representing protein impurities visible in the lanes of 0.1 M (wash) and 1 M NaCl (regeneration) fractions, but few protein impurities were visible in the elution step at 0.2 M NaCl. This step achieved an impurity protein clearance of 2.2 LRV and DNA clearance of 2.7 LRV (Table 3). This translated into an overall impurity protein clearance of 3.3 LRV and DNA clearance of 4.2 LRV in the two-step (CEX + AEX) membrane chromatography process, and a capsid recovery of the anion-exchange step of 74.8% (infective recovery of 86.3%). In addition, the combination of the CEX-IDA membrane and AEX-TEA membrane processes had a high infective recovery of 76.0%, which is higher than most reported results by ion-exchange chromatography [41,44,47]. The VG/capsids of the purified AAV2 from the AEX-TEA membrane step was 0.42, a substantial increase from the 0.26 obtained in the initial CEX-IDA membrane step. The morphological integrity of the purified viral particles was evaluated via transmission electron microscopy (TEM): the images reported in Figure 9 confirm the integrity and homogeneity of the product AAV2 compared to the viruses in the feedstock and the standard.

To evaluate whether a shorter residence time would increase the AAV2 recovery and purity, an RT of 0.1 min was employed for sample loading and the experiment was repeated. The chromatographic profiles of the resultant chromatograms (Figure S8A,B) were comparable to those obtained at the RT of 1 min (Figure 7a and Figure 8a), and the capsid recoveries were similar as well. However, the HCP LRVs obtained upon conducting the binding step at the RT of 0.1 min in both ion-exchange steps were lower than the counterparts obtained with an RT of 1 min (Table 3 and Table 4); the difference can be noticed in the SDS-PAGE analysis in Figure S8C, which shows a higher abundance of host cell proteins in the elution fractions obtained upon binding at the RT of 0.1 min, implying that the higher flow rate at 0.1 min RT enhanced the binding of protein impurities relative to the binding of capsids. One potential explanation for this effect is that the shorter residence times do not allow sufficient time for the viral particles to displace smaller impurity proteins that might bind to the surface.

## 4. Conclusions

Purification of viral vectors for gene therapy applications is one of the open challenges for the biomanufacturing industry. In this study, nonwoven ion-exchange membrane adsorbers were used for the first time for the capture and purification of AAV2 from crude Sf9 lysate. A new anion-exchange nonwoven membrane was developed (AEX-TEA) with a binding capacity for pure AAV2 of 9.6 × 10^13^ capsids/mL, a number which is significantly larger than those reported in the literature for other viral adsorbents. The viral particles can be eluted with high recovery using 0.2 M NaCl. A single bind-and-elute capture step with these AEX-TEA membranes showed higher capsid binding capacity and final purity when compared to a commercial benchmark. When exposed to a clarified Sf9 cell culture fluid producing AAV2, these nonwoven membranes were able to bind 2.4 × 10^13^ capsids/mL of membrane and achieved a host cell LRV of 1.8. Under identical loading and operating conditions, the commercial membrane Sartobind Q bound 1.9 × 10^13^ capsids/mL and exhibited a non-capsid protein LRV of 1.5. To improve the removal of empty capsids and the total impurity clearance, a two-step nonwoven membrane process was implemented: (i) pH 5 capture step with a cation-exchange CEX-IDA membrane, followed by (ii) pH 8 capture step using the anion-exchange membrane AEX-TEA. The combined process achieved a total capsid recovery of 65.3% with an infective recovery of 76%, values higher than those reported in the literature for ion-exchange chromatography. Moreover, the two-step process was able to obtain an overall impurity protein clearance of 3.3 LRV and a DNA clearance of 4.2 LRV. As for the empty capsids, a final value of VG/capsids of 0.42 was achieved that is a noticeable increase from the single-step process with VG/capsids of 0.2.

The present work demonstrates that integrated ion-exchange membrane chromatography processes can provide an efficient approach for the extraction of AAV viral particles from an Sf9 cell lysate and that nonwoven membranes have a great deal of promise for industrialization. Notably, the low cost of goods of these materials opens the door for potential single-use and flexible devices that can be applied to a wide variety of AAV serotypes and other viral vectors and cell lines in general.

## Figures and Tables

**Table 1:** Properties of the AAV2 feedstocks.

AAV2 Sources	AAV2 from Clarified Sf9 Cell Lysate	Pure AAV2 with Full Capsids (Standard)
Capsids concentration (capsids/mL) ^1^	1.1 × 10^13^	1 × 10^12^
VG/Capsids ^2^	0.19	>0.9
TU/mL ^3^	1.7 × 10^8^	2.2 × 10^7^
Protein concentration ^4^	~10 mg/mL	2.2 μg/mL

^1^ The number of total capsids, including full and empty capsids, was determined by ELISA. ^2^ The number of viral genomes (VG) was determined by Q-PCR, as detailed in Section 2.6. The ratio VG/capsids is the ratio of capsids with genome to total capsids. ^3^ Transducing unit (TU)/mL is defined as the number of cells expressing GFP per mL of injected AAV2 samples. The transduction assay is detailed in Section 2.6. ^4^ The total protein concentration was measured by Bradford assay.

**Table 2:** Separation performance comparison of AEX-TEA membrane and Sartobind Q. The non-capsid protein concentration and VG/capsids in the applied feed is 500 μg/mL and 0.19.

Membrane	AEX-TEA	Sartobind Q
Membrane type	Nonwoven	Microporous cast
Membrane volume (mL)	0.1	0.08
Specific surface area ^a^ (m^2^/g)	0.86 ^b^ [50]	0.90 [51]
Ligand density ^c^ (μmol/g)	600.0	553.6
Sample loading (capsids/mL of membrane volume)	2.5 × 10^13^	2.5 × 10^13^
% Capsids in FT ^d^	1.3%	25.8%
% Capsids in the 0.1 and 0.2 M NaCl elution fraction	87.9%	59.0%
Number of bound capsids/mL of membrane ^e^	2.4 × 10^13^	1.9 × 10^13^
Eluted capsid concentration (capsids/mL) ^f^	3.3 × 10^11^	1.9 × 10^11^
VG/Capsids ^g^	0.2	0.4
Non-capsid protein concentration (ng/mL) ^h^	4.4 × 10^3^ (1.8 LRV)	5.0 × 10^3^ (1.5 LRV)

^a^ Measured by Brunauer–Emmett–Teller (BET) nitrogen adsorption method. ^b^ Data of original PBT nonwoven. ^c^ Measured by nitrogen element analysis according to [38]. ^d^ Calculated as the ratio of the number of capsids in the flow-through fraction vs. feed. ^e^ Calculated as the number of capsids bound to the membrane. ^f^ Capsid concentration in the 0.2 M NaCl elution fraction for the AEX-TEA membrane and the capsid concentration in the 0.1 M NaCl elution fraction for the Sartobind Q membrane. ^g^ Calculated as the ratio of the number of viral genomes to total capsids (including the full and empty) in the elution fraction obtained at 0.2 M NaCl for the AEX-TEA membrane and at 0.1 M NaCl for the Sartobind Q membrane. ^h^ Calculated as the difference between the concentration of total protein and the concentration of the capsid-based proteins. The 0.2 M NaCl elution fraction was analyzed for the AEX-TEA membrane and the 0.1 M NaCl elution fraction was analyzed for the Sartobind Q membrane.

**Table 3:** Results of two-step AAV2 purification (CEX + AEX) at 1.0 min residence time (feed contained 6.0 μg/mL dsDNA, 170 μg/mL non-capsid protein concentration, and 0.19 VG/capsids).

	CEX-IDA Membrane (First Step)	AEX-TEA Membrane (Second Step)
% Recovery	1st Step capsid recovery: 87.3%	2nd Step capsid recovery: 74.8%
	1st Step infective recovery: 88.1%	2nd Step infective recovery: 86.3%
2-Step Combined Process	capsid recovery: 65.3%, infective recovery:76.0%	
Capsids concentration (capsids/mL)	1.1 × 10^11^	2.4 × 10^11^
VG/Capsids	0.26	0.42
Non-capsid protein concentration (ng/mL)	1.4 × 10^4^ (1.1 LRV)	78.1 (LRV: 2.2)
Contaminant dsDNA (ng/mL)	188.6 (1.5 LRV)	0.4 (LRV: 2.7)

**Table 4:** Results of two-step AAV2 purification (CEX + AEX) at 0.1 min residence time (feed contained 6.0 μg/mL dsDNA, 170 μg/mL non-capsid protein concentration, and 0.19 VG/capsids).

	CEX-IDA Membrane (First Step)	AEX-TEA Membrane (Second Step)
% Recovery	1st Step capsid recovery: 90.6%	2nd Step capsid recovery: 78.6%
2-Step Combined Process	capsid recovery: 71.2%	
Capsids concentration (capsids/mL)	1.2 × 10^11^	2.5 × 10^11^
VG/Capsids	N/A	N/A
Non-capsid protein concentration (ng/mL)	2.5 × 10^4^ (0.8 LRV)	636.4 (LRV: 1.6)
Contaminant dsDNA (ng/mL)	N/A	N/A

## Data Availability

The data presented in this study are available on request from the corresponding author.

## Supplementary Materials

The following supporting information can be downloaded at: https://www.mdpi.com/article/10.3390/membranes12100944/s1, Figure S1: Influence of reaction temperature on the SBC of AEX-TEA membrane; Figure S2: Typical chromatogram of DBC measurement of AEX-TEA membrane; Figure S3: Permeability of AEX-TEA membrane; Figure S4: BSA separation from IgG/BSA mixture by AEX-TEA membrane; Figure S5: Chromatogram of AAV2 separation by CEX-IDA membrane pH 5; Figure S6: Chromatogram of AAV2 separation by CEX-IDA membrane pH 5.5; Figure S7: Chromatogram of AAV2 separation by CEX-IDA membrane pH 6; Figure S8: Chromatogram of AAV2 purification by CEX-IDA membrane and AEX-TEA membrane at RT = 0.1 min and the corresponding SDS-PAGE; Table S1: DBC of AEX-TEA membrane at different RTs; Table S2: Results of AAV2 separation by CEX-IDA membrane at pH 5.0, 5.5, and 6.0. RT = 1.0 min; Table S3: Results of AAV2 separation by CEX-IDA membrane with step gradients of 0.1, 0.2, 0.3, and 1.0 M NaCl.
