# Supplementary material for: Purification of Adeno-Associated Virus (AAV) Serotype 2 from Spodoptera frugiperda (Sf9) Lysate by Chromatographic Nonwoven Membranes
Source: Membranes (Basel). 2022 Sep 27;12(10):944. doi: 10.3390/membranes12100944 (PMC9606886; doi:10.3390/membranes12100944)
Supplement: Supplementary file 1 [file membranes-12-00944-s001.zip › membranes-1930551-supplementary.pdf]

membranes

MDPI

Supplementary Material: Purification of Adeno-Associated Virus (AAV) Serotype 2 from *Spodoptera frugiperda* (Sf9) Lysate by Chromatographic Nonwoven Membranes

Jinxin Fan, Eduardo Barbieri, Shriarjun Shastry, Stefano Menegatti Cristiana Boi, Ruben G. Carbonell

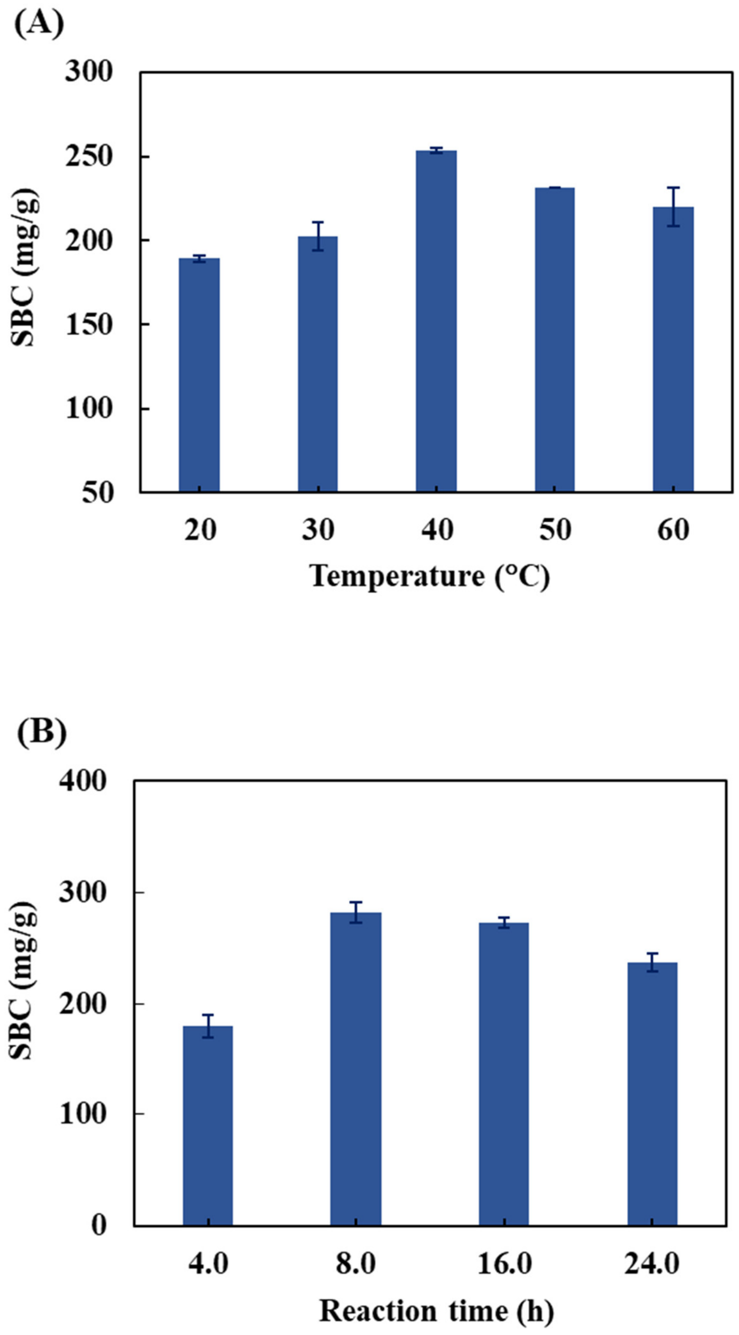

Figure S1. Influence of reaction temperature on the SBC of AEX-TEA membrane (**A**). Preparation conditions: 10% (*v*/*v*) TEA/water as reaction solution, TEA coupling for 2 h. Influence of reaction time on the SBC of

AEX-TEA membrane (**B**). Preparation conditions: 10% (v/v) TEA/water as reaction solution, TEA coupling at 40 °C.

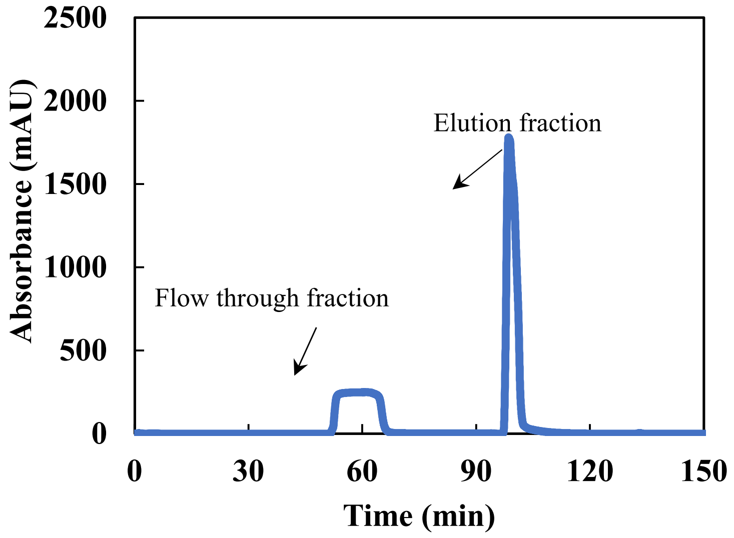

Figure S2. Typical chromatogram of DBC measurement of AEX-TEA membrane. 12 membrane layers were stacked in a 25 mm-diameter column. RT: 1.0 min. Preparation conditions: 10% (v/v) TEA/water as reaction solution, TEA coupling for 8 h.

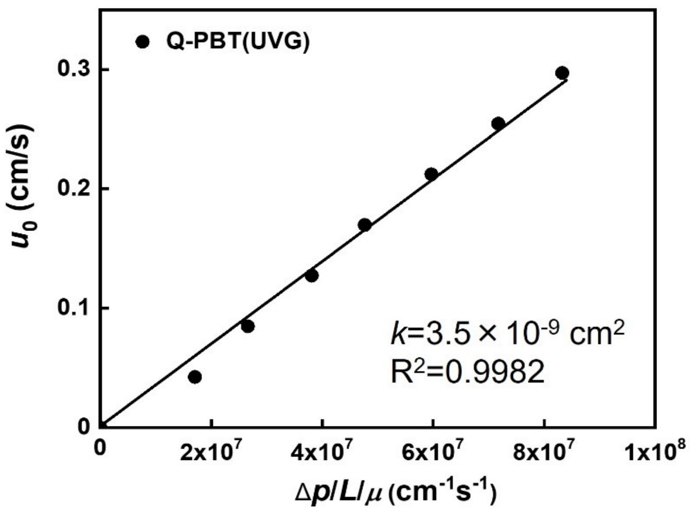

Figure S3. Permeability of AEX-TEA membrane in pure water at different superficail velocities (60 membrane layers in a 10 mm-diameter).

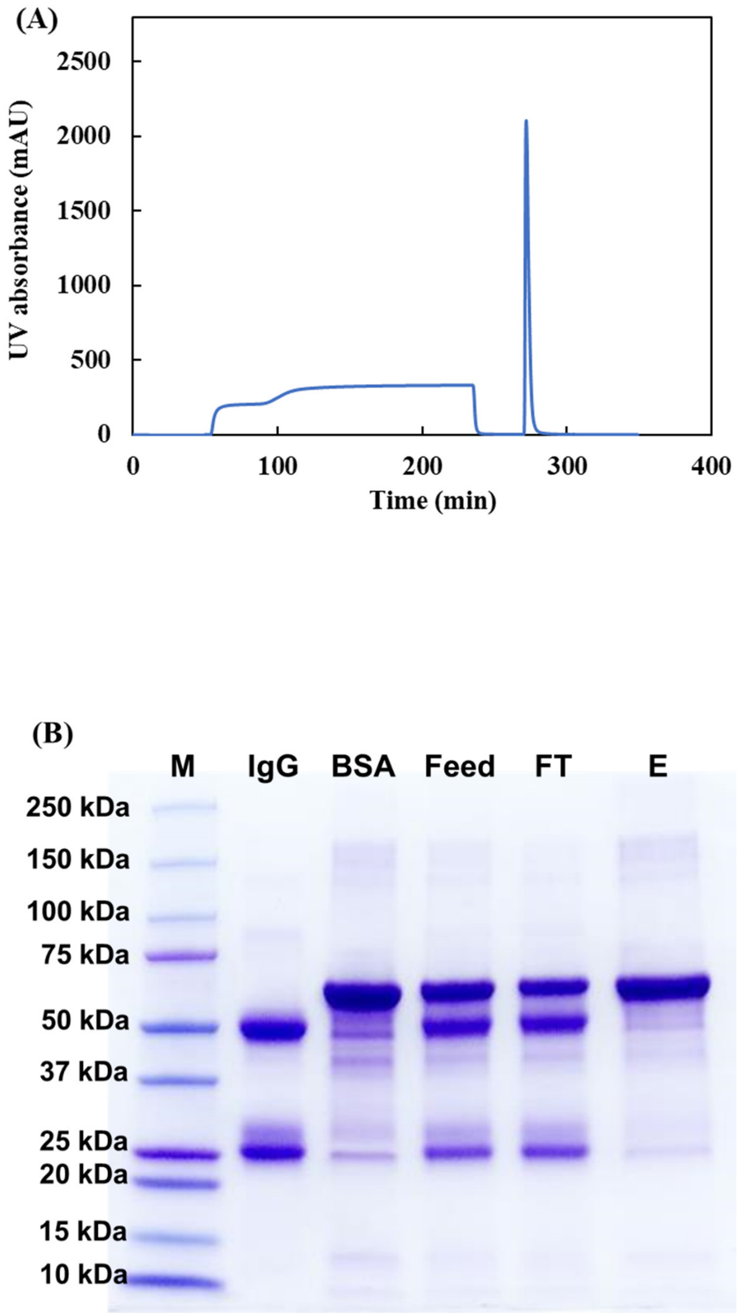

Figure S4. The chromatogram of BSA separation from IgG/BSA mixture by AEX-TEA membrane (**A**). SDS-PAGE characterization of BSA separation from IgG/BSA mixture. Lane M: molecular weight marker, lane IgG: IgG standard, lane BSA: BSA standard, lane Feed: IgG/BSA mixture, lane FT: flow through fraction, lane E: elution fraction (**B**).

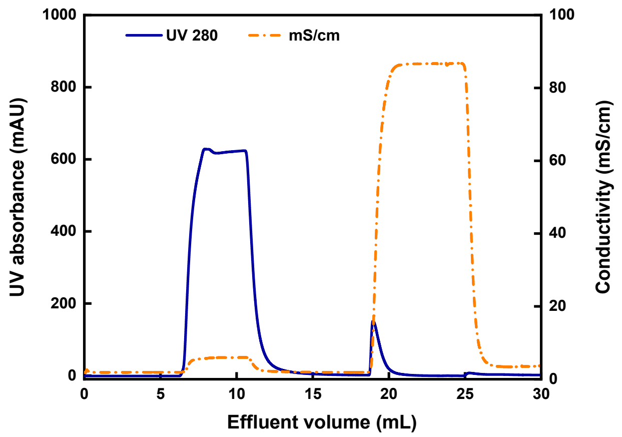

Figure S5. Chromatogram of AAV2 separation by CEX-IDA membrane. pH=5.0, RT=1.0 min, membrane volume=0.25 mL.

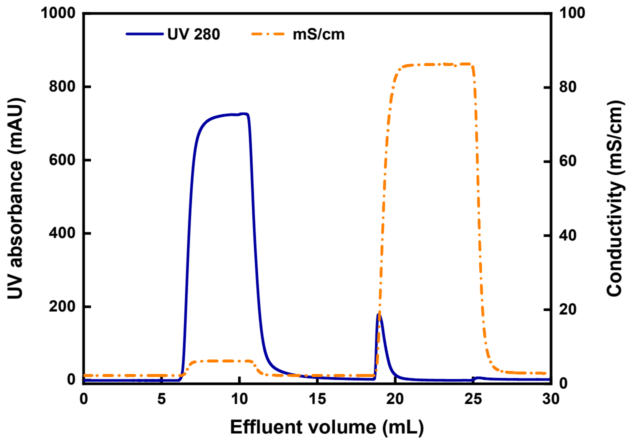

Figure S6. Chromatogram of AAV2 separation by CEX-IDA membrane. pH=5.5, RT=1.0 min, membrane volume=0.25 mL.

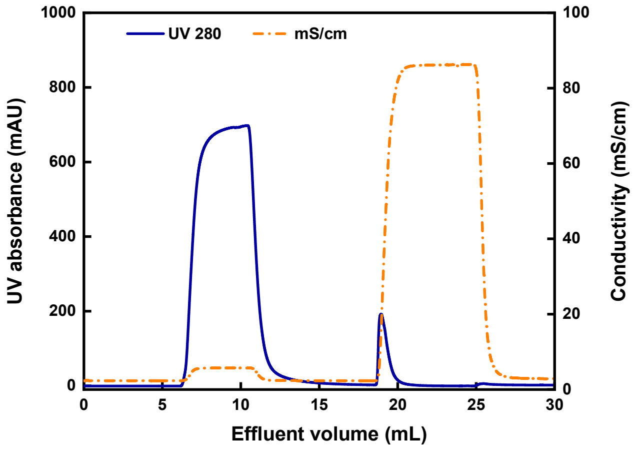

Figure S7. Chromatogram of AAV2 separation by CEX-IDA membrane. pH=6.0, RT=1.0 min, membrane volume=0.25 mL.

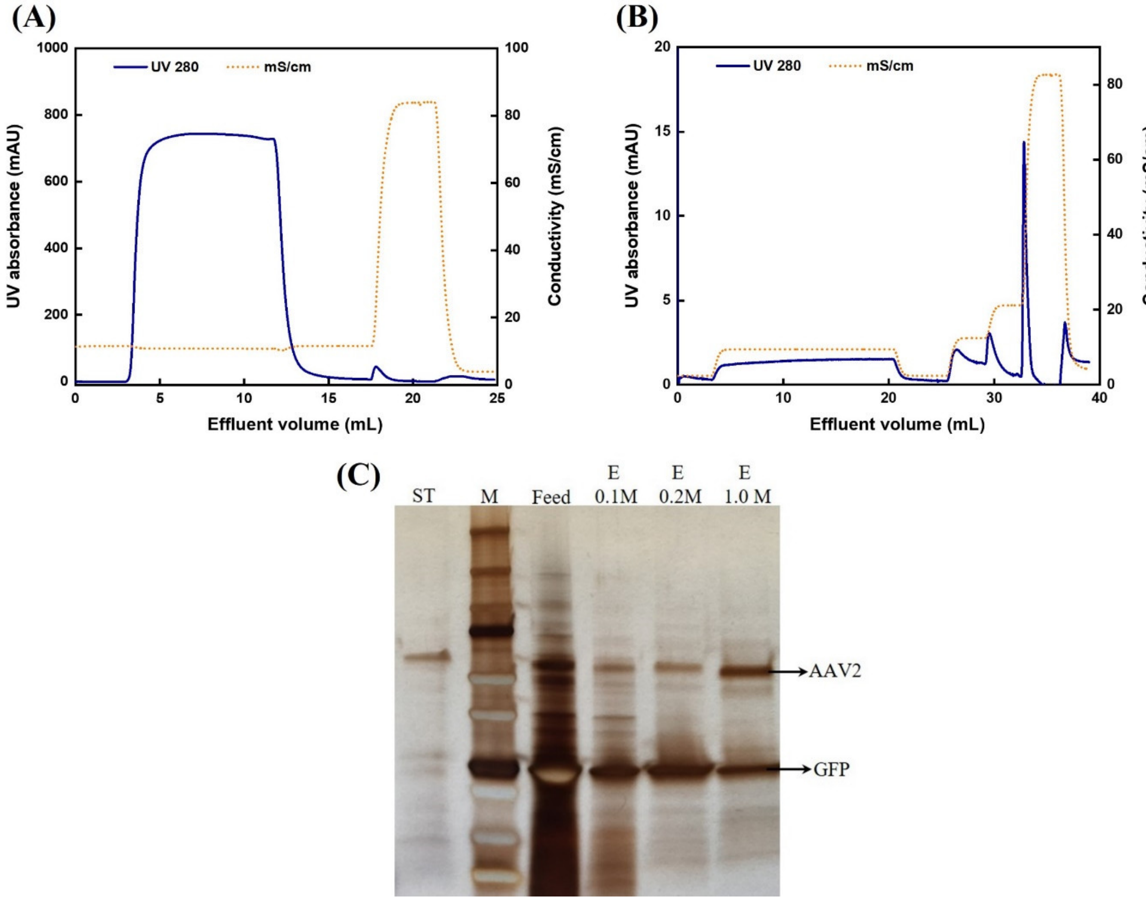

Figure S8. Chromatogram of AAV2 purification by CEX-IDA membrane (**A**) and AEX-TEA membrane (**B**) at RT=0.1 min and the corresponding SDS-PAGE analysis (**C**). "ST": standard pure AAV2, "M": marker; "Feed": 1.0 M NaCl elution fraction by CEX-IDA membrane; "FT": flowthrough; "E 0.1 M" and "E 0.2 M": elution with 0.1 M NaCl and 0.2 M NaCl buffer.

Table S1

DBC of AEX-TEA membrane at different RTs.

RT (min)	DBC (mg BSA/mL)
0.1	44.1
0.5	43.8
2.0	43.3

Table S2.

Results of AAV2 separation by CEX-IDA membrane at pH 5.0, 5.5 and 6.0. RT=1.0 min, membrane volume=0.25 mL. The number of capsids in the feed and flow through were quantified by ELISA and the number of the bound capsid is the difference between the loaded capsids and capsids in flow through. %Capsids is the number of capsids divided by the number of capsids in the feed.

Separation pH	% Capsids in FT	% Capsids bound on the membrane
5.0	0	100
5.5	24	76
6.0	100	0

Table S3.

Results of AAV2 separation by CEX-IDA membrane with step gradients of 0.1, 0.2, 0.3 and 1.0 M NaCl. pH=5.0, RT=1.0 min, membrane volume=0.25 mL. %Capsids was the number of capsids divided by the number of capsids in the feed.

	Feed	FT	0.1 M NaCl elution	0.2 M NaCl elution	0.3 M NaCl elution	1.0 M NaCl elution
Total capsids	4.5×10^{11}	Not detected	Not detected	1.2×10^{11}	1.3×10^{11}	1.3×10^{11}
% Capsids	100%	N/A	N/A	26.6	28.9	28.9
